# Persistent Multidrug‐Resistant *Salmonella enterica* Serovar Paratyphi A Bacteremia in a Preterm Neonate: A Case Report

**DOI:** 10.1155/crpe/2293042

**Published:** 2026-10-01

**Authors:** Khat Sophea, Srey Viso, Chean Sophâl

**Affiliations:** ^1^ Department of Neonatal Intensive Care Unit National Pediatric Hospital Phnom Penh Cambodia; ^2^ Department of Medical Laboratory National Pediatric Hospital Phnom Penh Cambodia; ^3^ Department of Pediatric Hematology and Immunology National Pediatric Hospital Phnom Penh Cambodia

**Keywords:** blood culture, multidrug resistance, neonatal sepsis, *Salmonella enterica* serovar Paratyphi A

## Abstract

**Background:**

Neonatal sepsis caused by *Salmonella enterica* serovar Paratyphi A is rare and may present with nonspecific clinical features. Multidrug‐resistant strains pose substantial diagnostic and therapeutic challenges, particularly in resource‐limited settings.

**Case Presentation:**

We report a preterm neonate born at 33 weeks of gestation with a birth weight of 1800 g who initially presented with respiratory distress and was managed as transient tachypnea of the newborn. The infant subsequently developed persistent fever, feeding intolerance, abdominal distension, leukopenia, severe thrombocytopenia, and elevated inflammatory markers despite initially negative blood cultures. A blood culture obtained on Day 20 of life grew multidrug‐resistant *S. Paratyphi A*, resistant to ceftriaxone, ciprofloxacin, pefloxacin, and trimethoprim–sulfamethoxazole, with intermediate susceptibility to amikacin. The infant required blood–product support and prolonged antimicrobial therapy. A 21‐day course of empiric cefoperazone/sulbactam plus levofloxacin was administered with close monitoring and was temporally associated with clinical improvement and microbiological clearance. Blood cultures obtained on Days 45 and 50 were negative. No maternal or apparent healthcare‐associated source was identified.

**Conclusion:**

Persistent multidrug‐resistant *S. Paratyphi A* bacteremia should be considered in neonates with refractory sepsis despite initially negative blood cultures. Repeated cultures, reassessment of antimicrobial susceptibility, and cautious individualized treatment are essential when laboratory capacity and therapeutic options are limited. The effectiveness of empiric salvage therapy cannot be established without direct susceptibility data and stronger supporting evidence.

## 1. Introduction

Neonatal sepsis caused by *Salmonella* species is rare but potentially life‐threatening. Its clinical manifestations are nonspecific and often indistinguishable from sepsis caused by more common neonatal pathogens, which may delay diagnosis and appropriate treatment. Globally, *Salmonella enterica* serovars Typhi and Paratyphi A remain important causes of invasive disease, particularly in low‐ and middle‐income countries.

The emergence of multidrug‐resistant (MDR) strains, including isolates resistant to multiple commonly used antimicrobial classes, poses a growing public health challenge [1]. In neonates, infection may occur through vertical transmission from the mother or horizontal transmission through contaminated food, water, caregivers, or the healthcare environment. Cases of neonatal sepsis caused by MDR *Salmonella enterica* serovar Paratyphi A (*S.* Paratyphi A) are uncommonly reported [2]. To the best of our knowledge, no such case has previously been reported from Cambodia. We describe a case of persistent MDR *S.* Paratyphi A bacteremia in a preterm neonate, highlighting its unusual resistance pattern, diagnostic challenges, and therapeutic implications in a resource‐limited setting.

## 2. Case Presentation

A preterm female neonate was born at 33 weeks of gestation with a birth weight of 1800 g by spontaneous vaginal delivery to a Gravida 2, Para 2 mother. She was initially admitted to the neonatology ward of a referring maternity hospital after birth. No prolonged rupture of membranes, maternal fever, gastrointestinal illness, intrapartum antibiotic exposure, or history of maternal enteric fever was documented. The infant cried immediately after birth, with Apgar scores of 7, 8, and 9 at 1, 5, and 10 min, respectively. Because of worsening respiratory distress at 18 h of life, she was transferred to the neonatal intensive care unit (NICU) of a tertiary pediatric referral center for further management.

On NICU admission, the infant had moderate respiratory distress, with a Silverman score of 5. Her temperature was 36.4°C, respiratory rate 50 breaths/min, heart rate 140 beats/min, and capillary refill time 2 s. Clinical signs included subcostal and intercostal retractions, nasal flaring, audible grunting, and mottled skin. Cardiac auscultation revealed normal heart sounds without murmur.

### 2.1. Investigations

Initial investigations included sepsis evaluation, chest radiography, and serum glucose measurement. Echocardiography was performed on Day 9 of life due to limited availability. Admission labs showed a total leukocyte count of 13.8 × 10^9^/L, absolute neutrophil count of 9 × 10^9^/L, 0% band forms (immature‐to‐total neutrophil ratio 0), platelet count of 253 × 10^9^/L, and C‐reactive protein (CRP) of 2 mg/L. Liver enzymes were mildly elevated (AST 98 U/L, ALT 34 U/L), while serum glucose, calcium, urea, and creatinine were normal. Chest radiography was unremarkable, and blood gas analysis was not performed due to equipment limitations. Based on the initial respiratory presentation and unremarkable chest radiograph, a provisional diagnosis of transient tachypnea of the newborn was made. Echocardiography revealed a 2.5‐mm patent ductus arteriosus with a nonrestrictive left‐to‐right shunt and a small patent foramen ovale, without other abnormalities. Intravenous paracetamol was started for PDA closure. Serial laboratory findings are summarized in Table 1.

**TABLE 1:** Serial laboratory findings during hospitalization.

Blood work	Day 0	Day 2	Day 8	Day 15	Day 20	Day 27	Day 34	Day 55	Reference range
*Complete Blood Count*									
WBC	13.8	9.9	9.9	6.5	4.5	6.6	5.8	3.9	10–26 × 10^9^/L
Neutrophils	9.0	5.7	2.4	2.2	1.8	3.3	2.9	1.7	4–14 × 10^9^/L
Lymphocytes	0	2.7	5.2	3.3	2.4	3.3	2.5	1.7	3–8 × 10^9^/L
Hemoglobin	20.9	16.7	18.4	12.4	8	8.2	9.6	10	14–22 g/dL
Platelets	253	192	241	8	2	2	4	134	100–450 × 10^9^/L

*Liver Enzymes*									
AST	98	52	34	21	18	23	50	337	< 31 U/L
ALT	34	17	20	11	16	17	19	160	< 32 U/L

*Renal Function*									
Urea	21	30	79	75	71	47	54	20	10–50 mg/dL
Creatinine	1.0	0.5	0.5	1.0	0.9	0.8	0.7	0.7	0.6–1.1 mg/dL

*Inflammatory Markers*									
CRP	2.0	2.0	3	48	48	24	48	1	< 6 mg/L

*Note:* ALT: alanine aminotransferase; AST: aspartate aminotransferase.

Abbreviations: CRP, C‐reactive protein; WBC, white blood cells.

### 2.2. Treatment

The infant was initially managed with nasal continuous positive airway pressure (CPAP) and empiric intravenous ampicillin, ceftriaxone, and gentamicin. CPAP was required for 4 days, followed by supplemental oxygen for 7 days, after which respiratory support was discontinued. Antimicrobial susceptibility testing was performed by disk diffusion and interpreted according to CLSI M100, 33rd edition (2023) [3]. No automated or MIC‐based method, including broth microdilution or E‐test, was available; therefore, MICs were not determined. Susceptibility testing was not performed for meropenem, cefoperazone/sulbactam, or levofloxacin. Routine quality‐control testing was conducted using standard control strains in accordance with laboratory procedures and CLSI recommendations.

During hospitalization, the infant developed intermittent fever, feeding intolerance, and abdominal distension from Day 8 of life. Serial blood cultures obtained at admission, Day 8, and Day 15 showed no growth. By Day 15, laboratory findings revealed progressive leukopenia, severe thrombocytopenia with a platelet count of 8 × 10^9^/L, and markedly elevated CRP of 48 mg/L (Table 1). The infant required repeated platelet transfusions and red blood cell transfusion for severe thrombocytopenia and anemia during hospitalization. Because of ongoing clinical and laboratory deterioration despite initial empiric therapy, antibiotics were escalated to ceftazidime and ciprofloxacin.

A blood culture obtained on Day 20 of life grew *Salmonella enterica* serovar Paratyphi A, which was confirmed by repeat culture on Day 21. The isolate was resistant to ceftriaxone and ciprofloxacin, both of which had already been administered, as well as pefloxacin and trimethoprim–sulfamethoxazole, with intermediate susceptibility to amikacin (Table 2). Meropenem susceptibility was not assessed because it was not included in the initial antimicrobial panel. Owing to persistent fever and clinical deterioration, antimicrobial therapy was empirically escalated on Day 24 to intravenous meropenem at 20 mg/kg/dose every 8 h and amikacin at 15 mg/kg/dose every 24 h. Although the blood culture obtained on Day 25 showed no growth, cultures on Days 31 and 35 remained positive. Azithromycin was included in the susceptibility panel for the first time on Day 31 and was reported as resistant. On Day 35, ciprofloxacin was reported as intermediate and amikacin as susceptible. Because susceptibility testing relied exclusively on disk diffusion without MIC determination or molecular characterization, these categorical differences should be interpreted cautiously and may reflect analytical variability.

**TABLE 2:** Blood culture results and antimicrobial susceptibility testing.

Day of life	Blood culture	Key antimicrobial susceptibility
0	No growth	—
8	No growth	—
15	No growth	—
20	*Salmonella enterica* serovar Paratyphi A	R: ceftriaxone, ciprofloxacin, pefloxacin, trimethoprim–sulfamethoxazole; I: amikacin
21	*Salmonella enterica* serovar Paratyphi A	I: amikacin; R: ceftriaxone, ciprofloxacin, pefloxacin, trimethoprim–sulfamethoxazole
25	No growth	—
31	*Salmonella enterica* serovar Paratyphi A	I: amikacin; R: azithromycin, ceftriaxone, ciprofloxacin, pefloxacin, trimethoprim–sulfamethoxazole
35	*Salmonella enterica* serovar Paratyphi A	S: amikacin; I: ciprofloxacin; R: azithromycin, ceftriaxone, pefloxacin, trimethoprim–sulfamethoxazole
45	No growth	—
50	No growth	—

*Note:* R: resistant; I: intermediate; S: susceptible. Cefoperazone/sulbactam susceptibility testing was not performed because of limited laboratory capacity. Azithromycin was not included in the initial susceptibility panel for the Day‐20 isolate and was first reported on Day 31. Meropenem was not included in the initial antimicrobial susceptibility panel for this organism.

In view of persistent bacteremia, the evolving susceptibility profile, and limited therapeutic alternatives, treatment was changed on Day 39 to intravenous cefoperazone/sulbactam at 35 mg/kg/dose every 12 h, expressed as the total combined product in a 1:1 ratio and equivalent to 17.5 mg/kg/dose each of cefoperazone and sulbactam, plus intravenous levofloxacin at 10 mg/kg/dose every 12 h. This combination was used as empiric salvage therapy with close clinical and safety monitoring. Cefoperazone/sulbactam susceptibility testing was unavailable, and direct levofloxacin susceptibility data were not obtained; therefore, levofloxacin was used cautiously as a last‐resort option. Fever resolved by Day 45, with improvement in feeding tolerance and abdominal distension. By Day 50, the infant was clinically stable and remained afebrile.

Blood cultures obtained on Days 45 and 50 were negative, and both agents were continued for 21 days, from Day 39 to Day 60. Elevation of liver enzymes was noted near the end of treatment and was monitored clinically; no clinical signs of hepatic failure were observed. Maternal evaluation, including complete blood count, CRP, blood culture, urine culture, and stool culture, did not identify a source of infection. No additional cases of *Salmonella* infection were identified in the NICU during this period, and no healthcare personnel reported symptoms suggestive of enteric fever. The infant was discharged on Day 60 of life at a weight of 2.5 kg, in good clinical condition, without fever or respiratory distress, and with adequate feeding tolerance. At outpatient follow‐up seven days later, laboratory testing showed a white blood cell count of 3.8 × 10^9^/L, an absolute neutrophil count of 1.44 × 10^9^/L, hemoglobin of 8.9 g/dL, platelet count of 132 × 10^9^/L, CRP of 2 mg/L, AST of 252 U/L, and ALT of 179 U/L. Because of anemia, a packed red blood cell transfusion was administered at 10 mL/kg. Repeat testing the following day showed a white blood cell count of 4.9 × 10^9^/L, hemoglobin of 12.1 g/dL, and platelet count of 146 × 10^9^/L. A further follow‐up visit was scheduled for 2 weeks after discharge to reassess the complete blood count and liver enzymes; however, the patient did not return and was subsequently lost to follow‐up. Blood culture results and antimicrobial susceptibilities are summarized in Table 2, and the clinical timeline is presented in Table 3.

**TABLE 3:** Timeline of clinical events, investigations, and results.

Day of life	Clinical findings	Investigations	Results/impressions
0	Birth; respiratory distress	CBC, CRP, blood culture, chest X‐ray	Blood culture negative
8	Intermittent fever, feeding intolerance, and abdominal distension	CBC, CRP, blood culture	Developing neutropenia; blood culture negative
9	—	Echocardiography	PDA 2.5 mm with nonrestrictive L‐to‐R shunt
15	Persistent low‐grade fever; feeding intolerance	CBC, CRP, blood culture	Leukopenia, severe thrombocytopenia (platelet 8 × 10^9^/L), markedly elevated CRP (48 mg/L); blood culture negative
20	Persistent fever; clinical deterioration	Blood culture	*Salmonella enterica* serovar Paratyphi A; R: ceftriaxone, ciprofloxacin, pefloxacin, TMP‐SMX; I: amikacin
21	Persistent fever	Blood culture	*Salmonella enterica* serovar Paratyphi A confirmed; I: amikacin; same resistance pattern
24	Persistent fever and clinical deterioration	—	Therapy was empirically escalated to meropenem and amikacin
25	Partial improvement	Blood culture	No growth
31	Persistent fever; thrombocytopenia	Blood culture	*Salmonella enterica* serovar Paratyphi A; I: amikacin; R: azithromycin, ceftriaxone, ciprofloxacin, pefloxacin, TMP‐SMX
35	Persistent positive culture	Blood culture	S. Paratyphi A; S: amikacin; I: ciprofloxacin; R: azithromycin, ceftriaxone, pefloxacin, TMP‐SMX
39	Switch to cefoperazone/sulbactam + levofloxacin (Day 1/21)	—	Empiric salvage therapy initiated; meropenem and amikacin discontinued
45	Fever resolved; improving	Blood culture	No growth
50	Clinical recovery; afebrile	Blood culture	No growth
60	Completed 21‐day salvage therapy; clinically stable	—	Antibiotics discontinued; discharged at 2.5 kg without fever or respiratory distress and tolerating feeds
67	Outpatient follow‐up	CBC, CRP, AST, ALT	Persistent anemia, mild leukopenia/neutropenia, and elevated liver enzymes; packed red blood cell transfusion administered
68	Posttransfusion reassessment	CBC	Hemoglobin increased to 12.1 g/dL; WBC and platelet count improved

*Note:* TMP‐SMX, trimethoprim–sulfamethoxazole; MDR, multidrug‐resistant.

Abbreviations: CBC, complete blood count; CRP, C‐reactive protein; L‐to‐R, left‐to‐right; PDA, patent ductus arteriosus.

## 3. Discussion

Persistent MDR *Salmonella enterica* serovar Paratyphi A bacteremia in a preterm neonate is rarely reported and poses particular diagnostic and therapeutic challenges. Most epidemiological studies of enteric fever focus on older children and adults, with neonates representing only a small proportion of reported cases [2, 4]. Population‐based incidence data specific to neonates are lacking, and the available evidence consists mainly of case reports and small series [2]. This rarity may partly reflect limited exposure to typical fecal–oral transmission routes during early life [5]. Cambodian surveillance data indicate that *S.* Typhi and *S.* Paratyphi A together accounted for only 0.3% of positive blood cultures from 2019 to 2023 [6]. To our knowledge, this is the first reported case of persistent MDR *S. Paratyphi A* bacteremia in a preterm neonate in Cambodia.

Despite its rarity, neonatal *Salmonella* infection can cause substantial morbidity. In a large US surveillance study from 1968 to 2015, 190,627 culture‐confirmed *Salmonella* infections were reported among infants; most presented with gastroenteritis, while bacteremia and meningitis occurred less frequently but were associated with higher hospitalization and case‐fatality rates [7]. Studies from endemic settings have similarly suggested that neonatal *Salmonella* infections are uncommon and may be underrecognized [8, 9]. These findings support the clinical importance of considering invasive *Salmonella* infection in neonates with persistent or refractory sepsis, particularly when routine empiric therapy fails.

The clinical presentation in this case was nonspecific and initially indistinguishable from other causes of neonatal sepsis. The infant developed persistent fever, feeding intolerance, abdominal distension, leukopenia, severe thrombocytopenia, and elevated inflammatory markers despite initially negative blood cultures. Severe thrombocytopenia and leukopenia were particularly prominent. Although nonspecific, these abnormalities are recognized manifestations of invasive salmonellosis and may result from bone marrow suppression, immune‐mediated platelet destruction, reticuloendothelial clearance, and endotoxin‐mediated platelet consumption [9–12]. Hemophagocytosis has also been described in typhoid‐associated pancytopenia [11]. Their persistence, together with ongoing fever and elevated CRP, supported repeated microbiological evaluation and may reflect the invasive nature of the infection and delayed microbiological diagnosis.

The source of infection remained unidentified despite maternal evaluation and NICU surveillance for possible nosocomial transmission. Neonatal *Salmonella* infection may occur through vertical transmission, exposure during delivery, contact with caregivers, contaminated feeding, or the healthcare environment [13]. However, as in several previous reports, no definitive source was identified in this case [14]. The delayed isolation of *S. Paratyphi A* after several negative blood cultures highlights the importance of repeated microbiological assessment when neonatal sepsis persists despite empiric therapy. Blood culture remains the diagnostic gold standard, and previous reports of neonatal *Salmonella* infection have likewise relied on culture confirmation to establish the diagnosis [12, 15, 16].

Microbiologically, the isolate showed resistance to multiple antimicrobial classes, including third‐generation cephalosporins, fluoroquinolones, macrolides, and trimethoprim–sulfamethoxazole, consistent with a MDR phenotype [1]. For clarity, the term MDR is used here to describe resistance across several clinically relevant antimicrobial classes and does not imply confirmation of the classical ampicillin–chloramphenicol–trimethoprim/sulfamethoxazole Salmonella MDR phenotype because ampicillin and chloramphenicol susceptibility results were unavailable. Resistance to pefloxacin is clinically important because CLSI M100 recommends pefloxacin disk testing as a surrogate marker for fluoroquinolone susceptibility in invasive Salmonella infections [3]. The difference in ciprofloxacin susceptibility categories should not be interpreted as evidence of an evolving resistance mechanism because MIC determination and molecular testing were unavailable. Azithromycin susceptibility was interpreted using CLSI breakpoints established for S. Typhi because dedicated criteria for S. Paratyphi A are not available in CLSI M100 (2023), representing an important microbiological limitation.

Management was challenging because the organism was resistant to several commonly used agents. Third‐generation cephalosporins are often used as first‐line therapy for invasive *Salmonella* infections; however, ceftriaxone resistance in this case limited their utility [17]. The isolate also showed resistance to ciprofloxacin, pefloxacin, azithromycin, and trimethoprim–sulfamethoxazole. Amikacin was initially reported as intermediate and subsequently as susceptible on Day 35. Although carbapenems are often considered for MDR *Salmonella* infections, meropenem susceptibility was not assessed because it was not included in the laboratory’s initial antimicrobial panel. This highlights the importance of expanding antimicrobial susceptibility testing when persistent bacteremia occurs despite sequential antimicrobial escalation, particularly in resource‐limited settings where laboratory panels may be restricted.

Because direct susceptibility testing for cefoperazone/sulbactam and levofloxacin was unavailable, the combination was used as empiric salvage therapy based on the evolving disk‐diffusion results, limited therapeutic alternatives, and clinical judgment. Cefoperazone/sulbactam has documented activity against some MDR Gram‐negative organisms, including Enterobacteriaceae, through β‐lactamase inhibition by sulbactam [18]. Evidence from neonatal intensive care settings has also described its use as a carbapenem‐sparing option for MDR nosocomial infections [19]. In the context of MDR *Salmonella*, cefoperazone/sulbactam has been reported to retain activity against a proportion of resistant isolates [20]. Levofloxacin was added cautiously because of persistent bacteremia and the absence of reliably active alternatives. Although fluoroquinolones are generally avoided in neonates, they may be considered for severe MDR infections when the expected benefits outweigh potential risks [21–24].

Neonatal *Salmonella* infection can be associated with substantial mortality, reported at up to 30% in some series [25]. This underscores the importance of early recognition, repeated microbiological testing, careful interpretation of susceptibility results, and optimal supportive care, particularly in resource‐limited settings. Positive blood cultures on Days 31 and 35 following a negative culture on Day 25 may represent persistent or intermittent bacteremia; relapse and strain relatedness could not be determined because molecular typing was unavailable.

## 4. Limitations

This case report has several limitations. Susceptibility testing relied solely on disk diffusion; MICs were unavailable, and meropenem, cefoperazone/sulbactam, and levofloxacin were not tested. Molecular testing and pharmacokinetic monitoring were also unavailable, limiting interpretation of resistance patterns, strain relatedness, and drug exposure. The infection source remained unidentified, and an occult focus could not be excluded. The timing of early blood cultures relative to antibiotic administration was uncertain. As a single case, the apparent response to salvage therapy cannot establish efficacy or be generalized to routine practice.

## 5. Conclusion

Persistent MDR *Salmonella enterica* serovar Paratyphi A bacteremia should be considered in preterm neonates with refractory sepsis despite initially negative blood cultures. Repeated cultures, careful interpretation of susceptibility results, and expanded antimicrobial susceptibility testing may help guide management. In this case, cefoperazone/sulbactam plus levofloxacin was used as empiric salvage therapy and was temporally associated with clinical improvement and microbiological clearance; however, the effectiveness of this regimen cannot be established from a single case without direct susceptibility data.

## Author Contributions

Khat Sophea and Chean Sophâl conceptualized the case report and drafted the manuscript. Srey Viso contributed to the microbiological interpretation and laboratory‐related content. Chean Sophâl critically reviewed, edited, and finalized the manuscript.

## Funding

This research received no specific grant from any funding agency in the public, commercial, or not‐for‐profit sectors.

## Disclosure

All authors approved the final version and agree to be accountable for the work.

## Ethics Statement

According to the institutional policy, formal ethics committee approval was not required for this single‐patient case report.

## Consent

Written informed consent was obtained from the patient’s parents for publication of this case report and any accompanying clinical information and images. Patient confidentiality was maintained, and identifying information was removed before manuscript preparation.

## Conflicts of Interest

The authors declare no conflicts of interest.

## Patient Perspective

The infant’s parents expressed gratitude for the care provided and relief at their child’s recovery after a prolonged hospitalization.

## Data Availability

The data that support the findings of this study are available from the corresponding author upon reasonable request.
